# First time referral reasons, diagnoses and 10-year follow-up of patients seen at a Dutch fast lane outpatient cardiology clinic

**DOI:** 10.1007/s12471-019-1266-x

**Published:** 2019-04-05

**Authors:** T. Lenderink, E. J. M. Balkestein

**Affiliations:** 1 Zuyderland Medical Center, Heerlen, The Netherlands; 2 Adelante Zorggroep, Hoensbroek, The Netherlands

**Keywords:** Cardiology, Rapid access, Outpatients, Long term follow-up

## Abstract

**Purpose:**

To describe reasons for referral, diagnostic procedures, diagnoses and long-term follow-up of first-time referred patients to a fast lane outpatient cardiology clinic (FLOCC).

**Methods:**

A descriptive report of results obtained in a newly organised outpatient clinic. Data up to final diagnosis were recorded from electronic medical records. Follow-up data were obtained from electronic medical records, contacting patients and/or their general practitioners.

**Results:**

During the first 3 months of 2007, 419 patients were seen at the FLOCC. Of these patients, 360 were referred by general practitioners, 55 by other specialists and four were self-referrals. The largest referral groups were: chest complaints (44%), palpitations (19%) and dyspnoea (12%). In 65% of the 419 patients, cardiovascular disease was ruled out and they were discharged. Of these, 41% of the diagnoses were made on the same day, with a further 44% after additional investigations, mostly Holter registration. During 10 years of follow-up, 49 patients died: 17 of cardiac, 29 of non-cardiac, mainly cancer, and three of unknown causes. Of the initially discharged patients, 35% were referred again after an average of 4 years (1,443 days), with 47% reporting similar complaints.

**Conclusion:**

Of the patients referred to our FLOCC, most had chest pain. In one-third of all patients, cardiac disease was ruled out on the same day. Of all the patients discharged, 85% were diagnosed after basic investigations that could be ordered by a general practitioner. Holter registration was the most frequently requested additional investigation. These results support the development of less expensive, easily accessible extramural cardiology clinics.

## What’s new?

- In this manuscript we publish information about reasons for referral and diagnoses in a non-acute cardiology setting generally not described in research papers as it concerns ‘day to day’ cardiology care.
- In 65% of the patients cardiovascular disease was ruled out and they were discharged.
- Ten year follow-up information for this group of patients.
- 36% return after discharge with the same or other complaints.
- The main cause of death after 10-year follow-up was cancer, regardless of the diagnosis or whether the patient was discharged or not.
- These data could help in developing easily accessible, less expensive extramural outpatient care.

## Introduction

Daily work at cardiology outpatient clinics consists mainly of follow-up consultations for known cardiac disease. A minor part of the consultations involve newly referred patients with suspected cardiac disease. The Zuyderland Medical Center location Heerlen is a regional non-academic hospital covering an area of 250,000 inhabitants in the southern part of the Netherlands. Until 2007, it was common practice that patients newly referred to the cardiology outpatient clinic were first seen by a cardiologist. After this consultation, additional investigations, if required, were planned over the following weeks. Investigations could be, for instance: blood sampling, exercise testing; echocardiography, Holter registration, chest X‑ray and other invasive or noninvasive testing. Together with the existing average waiting period of 4 weeks for the first appointment, the above-mentioned procedure resulted in a period of several weeks before a diagnosis and a possible treatment proposal was available. Based on experiences from the rapid access cardiology units in the UK [1], a fast lane outpatient cardiology clinic (FLOCC) was introduced at the end of 2006, aiming for a complete diagnostic workup within one day. With this observational study we describe our experience with the FLOCC. This study describes reasons for referral, choice of diagnostic tests and the initial outcome of this fast diagnostic procedure, as well as re-referral rate and survival during 10-years of follow-up.

## Methods

After approval by the hospital’s Medical Research Ethics Committee, data were collected and analysed from electronic medical records (EMRs) for all first time patient referrals to the FLOCC during the first quarter of 2007. First time patient referral was defined as a consultation for a patient who had never before been seen by a cardiologist. Patients were referred either by general practitioners (GPs) or specialists for any complaints of suspected cardiac origin, except those patients suspected of having acute cardiac pathology, who are normally referred directly to the emergency department.

After initial referral, patients were seen within 5 working days by the cardiologist with an electrocardiogram (ECG) together with recent blood results, if available. During the consultation, assessment was done by history taking and performing a physical exam. In the following 3 hours, additional investigations were performed if warranted, such as echocardiogram, exercise test and/or chest X‑ray. Patients were then seen again that same day by the same cardiologist. Results were discussed, and a diagnosis was either made or postponed awaiting results from additional investigations, such as Holter registration in case of palpitations.

Reasons for referral were, based on frequency, categorised into four groups: chest pain, palpitations, dyspnoea and miscellaneous (sudden loss of consciousness, abnormal ECG, screening for cardiac disease, murmur, preoperative screening and cardiac origin of emboli).

Information at 10-year follow-up regarding survival, re-referral and diagnosis was primarily collected by consulting EMRs. If data were lacking within the 10-year period, patients were contacted personally. When this was not possible, GPs were approached by phone.

Due to the explorative design of the study, descriptive statistics are used for the results. Patient characteristics were summarised as mean (SD) or median (IQR) for continuous variables and percentages for categorical variables. A body mass index (BMI), ranging from 25.0 to 29.9 kg/m^2^, was defined as overweight.

## Results

From 2 January until 31 March 2007, a total of 445 patients were seen. Twenty-six were excluded from this study because they were not true first-time referrals. Of the remaining 419 patients, 194 (46%) were male. Median age was 57.3 (IQR = 20) years. Age ranged from 17–85 years in male patients and 17–91 years in female patients. A total of 360 patients were referred by GPs, 55 by other specialists and four were seen on self-referral.

Reasons for referral are given in Tab. 1. The main reason for referral was chest pain, followed by palpitations (Tab. 1). Most patients (74–84%) among the four referral groups did not have a medical history (Tab. 2). A small number of patients in each group had cardiac risk factors such as hypertension (30–42%), hypercholesterolaemia (12–22%), diabetes (6–15%) or smoking (12–24%). Overweight was present in three of the four groups. Family history for cardiovascular disease, as opposed to family history for sudden cardiac death, was frequently reported in all four groups, ranging from 32% in the dyspnoea group to 46% in the chest pain group. Peripheral arterial disease and a history of cerebral vascular disease (transient ischaemic attack and cerebrovascular accident combined) were reported in a maximum of 13% of the patients in each group (Tab. 2).

**Table 1:** Reasons for referral

	male (*n* = 194) (%)	age (mean)	female (*n* = 225)	age (mean)
chest pain	95 (49%)	56	90 (40%)	58
palpitations	23 (12%)	54	58 (26%)	49
dyspnoea	22 (11%)	62	28 (12%)	68
sudden loss of consciousness	14 (7%)	58	11 (5%)	52
abnormal ECG	16 (8%)	58	7 (3%)	68
screening for cardiac disease	12 (6%)	49	8 (4%)	54
murmur	5 (3%)	63	11 (5%)	65
preoperative screening	5 (3%)	66	9 (4%)	61
cardiac origin of emboli	2 (1%)	64	3 (1%)	49

**Table 2:** Baseline characteristics per main referral group

	chest pain (*n* = 185)	palpitations (*n* = 81)	dyspnoea (*n* = 50)	miscellaneous ^a^ (*n* = 103)
male (%)	95 (51%)	23 (28%)	22 (44%)	54 (52%)
median age (range)	57 (21–87)	51(17–85)	65(36–91)	58(17–85)
no medical history	146 (79%)	68 (84%)	38 (76%)	76 (74%)
PAD	12 (6%)	4 (5%)	4 (8%)	13 (13%)
TIA/CVA	8 (4%)	4 (5%)	2 (4%)	12 (12%)
family history of CVD	86 (46%)	28 (35%)	16 (32%)	40 (39%)
family history of SCD	9 (5%)	4 (5%)	2 (4%)	7 (7%)
hypercholesterolaemia	29 (16%)	11 (14%)	11 (22%)	12 (12%)
hypertension	55 (30%)	25 (31%)	21 (42%)	32 (31%)
diabetes	26 (14%)	5 (6%)	7 (14%)	15 (15%)
current smoker	45 (24%)	18 (22%)	6 (12%)	13 (13%)
BMI (median, IQR)	26.4 (5.6)	24.3 (6.0)	27.7 (7.1)	25.9 (6.5)

*PAD* peripheral arterial disease; *TIA* transient ischemic attack; *CVA* cerebrovascular accident; *CVD* cardiovascular disease; *SCD* sudden cardiac death; *BMI* body mass index.; *IQR* interquartile range.

^a^ Sudden loss of consciousness, abnormal ECG, screening for cardiac disease, murmur, preoperative screening and cardiac origin of emboli

After completion of all investigations no exact diagnosis could be given to 14 patients (3.3%), either because they did not attend for the additional investigation (eight patients) or a definite diagnosis was not given by the cardiologist (Tab. 3).

**Table 3:** Visits needed for final diagnosis

	total (*n* = 419)	1 day/visit *n* = 211; (% of total)	2 visits *n* = 182 (% of total)	>2 visits *n* = 20 (% of total)
no cardiovascular disease	274	137 (50)	124 (45)	13 (5)
coronary artery disease	35	14 (40)	18 (51)	3 (9)
cardiomyopathy	25	15 (60)	8 (32)	2 (8)
valvular disease	25	21 (84)	4 (16)	–
hypertension	22	13 (59)	9 (41)	–
arrhythmia	18	5 (28)	11 (61)	2 (11)
orthostasis/collapse n.o.s.	6	–	6 (100)	–
no diagnosis or lost to follow-up^a^	14			

Final diagnosis was reached after 1 visit (equals 1 daypart, third column), 2 visits (fourth column) or more than 2 visits (fifth column)

*n.o.s.* not otherwise specified

^a^ 8 patients did not show up at or could not perform their additional investigations so no final diagnosis could be made; 6 patients were completely lost to follow-up.

**Fig. 1:**
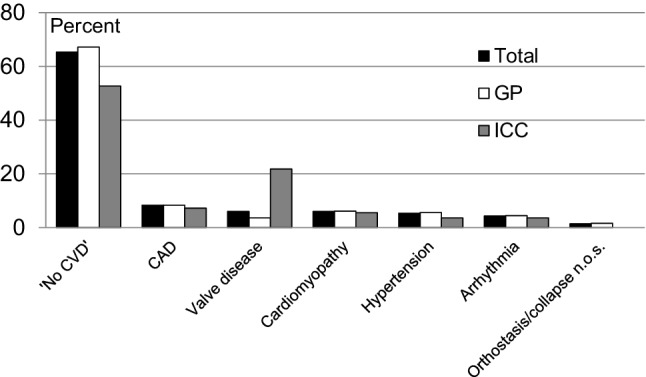
Diagnoses given after completion of all investigations. ‘*No CVD*’ no cardiovascular disease; *CAD* coronary artery disease; *n.o.s.* not otherwise specified; *GP* referred by general practitioner; *ICC* seen in consultation for other specialist

In 274 patients (65%) cardiovascular disease was ruled out (‘No CVD’). The most common diagnosis of cardiovascular origin was coronary artery disease, followed by cardiomyopathy and valve disease (Fig. 1). Valve disease was more often diagnosed in patients seen in consultation by other specialists than for GPs.

At times, multiple visits were needed to attain a final diagnosis (Tab. 3). The diagnosis of ‘No CVD’ could be reached in 50% of cases within the same day, an additional 45% were diagnosed after one additional visit to the cardiologist at the outpatient clinic on a later date. In valve disease, a diagnosis was made in 84% of patients during the first visit, in contrast to the diagnosis of arrhythmias, which was given in 28% of cases on the first visit (Tab. 3).

**Fig. 2:**
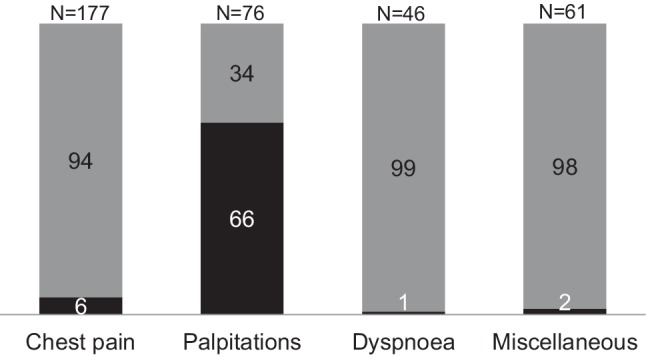
Patients (*n* = 360) referred by general practitioners, shown by referral group. Black part shows percentage where only an additional Holter registration was done for reaching a final diagnosis

The most ordered additional investigation was Holter registration. In 50 of 76 patients referred for palpitations by their GP, only a Holter registration and a follow-up visit to the cardiologist was needed for a final diagnosis. In the other referral groups, only an additional Holter registration was done in 1–6% of cases (Fig. 2).

**Fig. 3:**
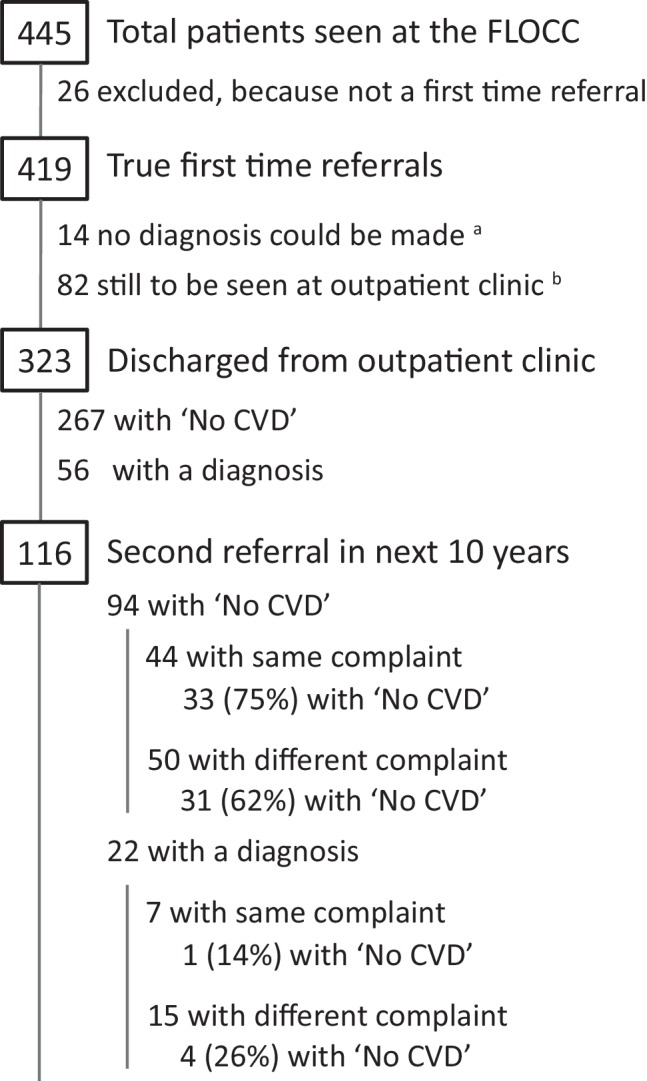
Flow of patients referred to the FLOCC. ‘*No CVD*’ no cardiovascular disease. ^a^ 8 patients did not show up at or could not perform their additional investigations so no final diagnosis could be made; 6 patients were completely lost to follow-up. ^b^ 75 patients with a diagnosis and 7 with ‘No CVD’

Of the 419 patients, 82 received a diagnosis of cardiac disease and were followed up by a cardiologist at the outpatient clinic. For 14 patients a diagnosis could not be established because they did not return for additional investigations or follow-up. The remaining 323 patients were discharged to their GP. Of these 323 patients, 267 were discharged with ‘No CVD’ and 56 with a diagnosis (Fig. 3).

Within a 10-year period, 116 (36%) of the discharged patients were again referred after an average of 1,443 days: 94 (35%) patients from the ‘No CVD’ group and 22 (39%) from the group with a diagnosis. Of those referred again with an initial ‘No CVD’ diagnosis, 44 patients presented with the same complaint. A ‘No CVD’ diagnosis was again given to 33 out of 44 patients (75%). Fifty were referred for another complaint. Of those, 31 patients (62%) again received the diagnosis of ‘No CVD’ (Fig. 3).

As mentioned above, 22 patients from the discharged group with a diagnosis were again referred. Of those, seven patients with the same complaint and 15 with a different complaint. Of the latter group, four were diagnosed ‘No CVD’. Of those referred with the same complaint, one was diagnosed as ‘No CVD’ (Fig. 3).

**Fig. 4:**
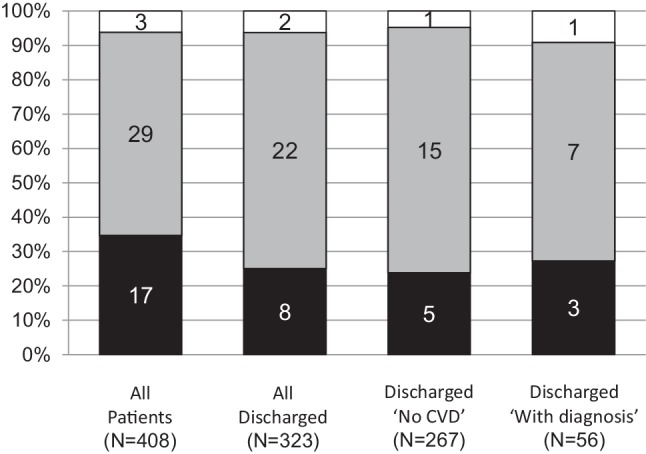
Causes of death during 10-year follow-up presented in percentage. In columns the absolute numbers are given (cardiac = *black*, non-cardiac = *grey*, unknown = *white column*) for all patients, all discharged patients, discharged with ‘No CVD’ and discharged with a diagnosis. ‘*No CVD*’ no cardiovascular disease

Ten year follow-up data were complete for 408 out of 419 cases. Forty-nine patients died (12%). Cancer was the main cause of death, being 20 out of 29 non-cardiac deaths (Fig. 4).

Of all 323 originally discharged patients, 32 had died (9.9%). In eight of these cases (25%), the cause of death was of cardiac origin.

Of the 267 discharged with ‘No CVD’, 21 (7.8%) died. Of these 21 patients, five died from a cardiac cause (25%). Of the 11 deceased patients in the group discharged with a diagnosis, three (25%) died from a cardiac cause (Fig. 4).

## Discussion

To decrease waiting time and thereby increase patient satisfaction, rapid access outpatient clinics have been established in the United Kingdom, with a first description of this health care model in 1976 [2]. In order to reduce waiting time for an appointment, investigations and final diagnosis, a fast lane outpatient cardiology clinic (FLOCC) was established in the southern province of the Netherlands in 2006. The first rapid access clinics in the United Kingdom were predominantly intended for the evaluation of chest pain [3] and a few for either heart failure or arrhythmia [1, 4, 5]. In contrast, at the FLOCC all patients suspected of having complaints of cardiac origin were accepted.

The main reason for referral in our study was chest pain. Palpitations were the second most stated reason for referral, with dyspnoea third. The distribution of reasons of referral was similar to the distribution described by Falces et al. [6]. However, in our study, murmur was probably underrepresented as a reason for referral because of the possibility for GPs to refer for an echocardiogram only [7]. In patients referred by other specialists, however, the group with valve disease was larger, probably because a murmur was heard during preoperative screening.

Results show that in two-thirds of the patients, cardiovascular disease was ruled out (‘No CVD’). In the Klimis review [3], comparable results are presented. It should be noted however, that the studies mentioned in this review were for chest pain patients only, and the criteria for referral were diverse and ranged from those using obligatory forms for the GP to referral from emergency departments.

Although not studied in our study or to our knowledge in any other study, we hypothesise that patient satisfaction is enhanced when they are diagnosed as soon as possible, preferably within 4 hours. In our study, one out of two patients could be discharged with a diagnosis within 4 hours or less. In order to discharge from follow-up, one additional visit to a cardiologist was needed in 45% of cases. Most of these cases were patients referred for palpitations for which only an additional Holter registration was needed. Based on these results, the time to a final diagnosis could be shortened by doing a Holter registration before the actual referral for palpitations.

Rathod et al. [8] found that using a ‘rapid access chest pain clinic referral form’ improves the quality of referrals. In our opinion, the use of such a form could also reduce the burden of referrals to the outpatient clinics in our setting.

We presume that fewer patients would have been referred to the outpatient clinic at the hospital if GPs had had access to diagnostic tests with interpretation by a cardiologist. This would possibly result in a population seen at the outpatient clinic with a higher a priori chance of cardiovascular disease and a higher number of patients diagnosed with cardiovascular disease.

It appears worthwhile to better predict the probability of having cardiovascular disease. This could result in keeping low-risk patients in the GP setting and sending those patients with a high chance of cardiovascular disease to the outpatient clinic. With this idea in mind, GPs in our region were given the opportunity to create a GP based and owned extramural clinic, a so called ‘anderhalvelijnszorg centrum’, an interface between primary and secondary care where basic simple secondary care investigations can be performed and interpreted by a cardiologist. Final results are then presented to the GP or directly to the patient. If warranted, patients can then be referred to the outpatient cardiology clinic at the hospital.

In future studies, it has to be investigated whether better risk stratification at the above mentioned, GP based and owned extramural clinic, will result in lowering costs for health insurance companies, patients and general society and furthermore enhance patient satisfaction. As a consequence, costs for hospitals might be higher as this strategy will lead to referral of a higher proportion of high-risk patients with, consequently, a higher number of more expensive investigations.

To our knowledge, this is the first study to report long-term follow-up of all kinds of first time referrals to a cardiologist. Especially follow-up data on discharged patients with a diagnosis of ‘No CVD’ are lacking. Follow-up at 10 years (the longest period described thus far) showed that one-third of all patients returned with the same or a new complaint. This number did not differ between those diagnosed with ‘No CVD’ and those with a diagnosis. Whether this is reassuring is debatable, but again two-thirds of the ‘No CVD’ patients did not have cardiovascular disease. These results are in line with current data, although with much shorter periods of follow-up [4]. With regard to cardiac causes of death, in patients classified as low risk (i. e. those visiting the outpatient clinic in contrast to the emergency department), our results are in accordance with published data [4, 9, 10]. The main cause of death at 10-year follow-up, in patients originally referred for suspected cardiac disease, was cancer.

In conclusion, two-thirds of the patients referred to our FLOCC could be diagnosed within two visits and the majority had ‘No CVD’. In one-third of all patients cardiac disease was ruled out on the same day. Of all the patients discharged, 85% were diagnosed after investigations nowadays easily available to the general practitioner. Holter registration was the most frequently requested additional investigation. A Holter registration ahead of consulting the cardiologist in cases of palpitations will reduce the time to a final diagnosis by 1 day. The main cause of death after 10-year follow-up was cancer, regardless of diagnosis and whether the patient was discharged or not.
